# Rethinking Restriction in Residential Aged Care: Dis/Abling Movement and Relations in the Time of COVID-19

**DOI:** 10.1177/12063312231159227

**Published:** 2023-03-09

**Authors:** Angela R. Y. Zhang, Tanya Zivkovic

**Affiliations:** 1 The University of Adelaide, South Australia, Australia

**Keywords:** care, aging, assemblage, COVID-19, movement, restriction

## Abstract

Restricting movement is a major focus in policy directives to reduce the spread of COVID-19 in aged care homes. In this article, we rethink dominant framing of restriction through a critical examination of the politics of good care and ethnographic attention to spatial extensions and interdependencies between residents, care workers, and assistive technologies. Drawing on ethnographic observations in two South Australian care facilities, analysis of aged care policies and national inquiries into aged care, and relevant media reporting, we examine how restriction to movement, misconceptualized as a good form of care, has suppressed residents’ physical and social needs and ruptured abling assemblages of resident mobility. We propose that walking alongside aged and frail residents offers new ways for thinking about care and re-abling relational approaches to care in times of crisis.

## Introduction

Movement is a central theme in these pandemic times. Even the motility of a virus is dependent on the movement of its hosts. Without bodies carrying infection, the transmission of COVID-19 would stop. However, moving is a core component of our social life. Bodies move in order to care for others and to be cared for, to reach out and assist, and to make one’s way in the world. Decisions about movement become particularly complicated and contested in settings such as residential aged care facilities, where residents’ bodies often depend on the movements of others who must assist them to move. In these spaces, the bodies of residents and care workers must conjoin to enable movements that neither aged and frail bodies nor viruses could make on their own.

Movement is thus a persistent concern in the lives of aged care residents and those who care for them. Having falls, or losing the ability to walk or move safely, is what brings most people into residential aged care; however, ethnographic observation of residents’ everyday lives revealed a more nuanced picture of movement (and its restriction) in care settings. Residents’ repeated attempts to stand up from sitting could be seen as demonstrative of a desire to walk and a need for assistance to do so. Yet these same movements, when observed by nurses in the context of clinical assessment, could be interpreted as “impaired balance” compounded by “confusion,” indicating a “loss of mobility” and heightened “risk of falls.” While residents exhibit a wide range of physical abilities, aged care discourse often describes their movements using terms like “exercise of the physically impaired” or the “risky behavior” of residents living with dementia. Corresponding to these multiple interpretations of movement, daily care activities were organized at times as “mobility care” and, at other times, as “fall prevention”: Such movement could be either encouraged and enabled or, conversely, forcibly restrained. The basis of COVID-19 health policy, primarily informed by epidemiological discourse, was that movement generated virus transmission, and thus, the most effective infection-control measures in aged care facilities would be ones that minimized movement. Under this policy directive, restrictive practices were strictly implemented across Australia, which resulted in the confinement of residents in their rooms.

In this article, we examine the complex lived realities of aged care homes before and during the COVID-19 pandemic. We draw on the work of Mol (2008) to consider what constitutes good care, exploring the disjunctures between residents’ needs to move and the politicization of care. Central to this analysis is our positioning of movement as vital to the social worlds of residents. The issue of mobility is not just about the individual and their ability or inability to move. In line with Schillmeier (2007), we also attend to the ordinary acts and materialities of everyday life that make up the multiple lived scenarios of older persons with disabilities. Schillmeier suggests that notions of disability and ability cannot be separated; thus, dis/ability should be seen as “complex sets of heterogeneous practices that (re-)associate bodies, material objects, and technologies with sensory practices” (Schillmeier, 2007, p. 195). Foregrounding these ideas, we examine how bodies, especially as they age, are always dependent upon multiple persons and entities to live and to get around in the world. We use the Deleuzian concept of assemblage to analyze how residents’ bodies entwine with this multiplicity—of persons, diseases, policies, viruses—involved in the delivery of everyday care. In this way, joining Schillmeier’s (2007) notion of dis/ability with the Deleuzian concept of assemblage (Deleuze & Guattari, 1988) we consider resident im/mobility in the context of the complex and shifting interplay between bodily impairments, material surroundings, aged care discourses, and policies to create what we term dis/abling assemblages.

Some assemblages operate to assist residents, while others constrain bodies, preventing movement and limiting the sensorial and spatial dimensions of residents’ worlds. Restrictive practices are entrenched in aged care homes, and their use has been amplified by COVID-19. From restraint technology such as sensor mats and “princess chairs” to workplace policies that prioritize fall prevention over mobility care, residents’ surroundings are specifically designed to limit movement. Examining the multiple and shifting dis/abling assemblages of resident movement, we think about care with science and technology studies (STS) scholars (Martin et al., 2015; Mol, 2002, 2008; Puig de la Bellacasa, 2011) and ask what is or is not assembled and why? Attending to the complex entanglements of policies, practices, needs, desires, resources, and interests, we show that the political dimensions of care come to define the movement policy for residents. The COVID-19 emergency policy has now extended restriction to movement in many ways, meaning that assemblages that previously enabled residents to move have been severely dismantled.

This article critically examines the notion of good care (Mol et al., 2010; Schillmeier, 2014) by tracing the movements—and restrictions—of aging bodies and care workers before and during the pandemic. First, it draws on a pre-COVID-19 ethnographic study of two South Australian nursing homes to illustrate some residents’ experiences of aging, how it affected their bodies and movements, and the generative capacity of certain relational interdependencies. When residents struggled to make their bodies do the things they wanted to do, enabling assemblages were made possible in everyday spatial and sensory extensions of residents’ bodies and their engagements with other people and things (Dennis, 2007; Latimer & Munro, 2009; Petty, 2021). From the touch of carers to well-placed handrails and walking aids, residents’ bodies were able to move and to walk. Good care became apparent as residents related to others who responded and enabled them to move safely by providing an assemblage of attention, aids, and assistance. This form of good care, advocated in quality standards and policies, was certainly perceived by staff as ideal but sadly was provided only occasionally.

The emergence of COVID-19 in Australian residential aged care facilities in 2020 led to a constellation of restrictive practices that totally severed residents’ sensory and spatial interconnections with the world: With the banning of visitors and confining residents to their rooms, there was an extreme absence of touch. A “back-to-basics” policy was applied that short-circuited the normal distribution of care, rupturing residents’ connections with the world outside the facility. This could be seen as a rhetorical veneer used by administrators to legitimize reductions in resources and staff. With fewer hands on deck, there were fewer resources available with which to assemble good care for residents.

This article is based on 12 months of ethnographic fieldwork in two South Australian care facilities (2015–2016) and a discourse analysis of aged care policies, reports, and national inquiries into aged care before and during the pandemic, supplemented with COVID-19-related reporting in news and social media from 2020 to 2022. Carrying out participant observation in the nursing homes, Zhang (2023) regularly walked with residents and observed their day-to-day interactions with care staff, family members, and assistive devices. In walking with residents, Zhang came to witness how residents lived in tension between moving and not moving and how they relied on multifaceted interdependencies involving people and technologies to move and to walk (Zhang, 2023). Insights gained from fieldwork on resident movement guided the discourse analysis and led to a rethinking of restriction to movement both in relation to and beyond COVID.

## Dis/Abling Assemblages

Difficulty with moving is strongly related to admission into residential aged care, and such were the cases of residents we met in the care homes. In this section, we turn to these residents and to their experiences of moving and aging. Residents spoke to the ethnographer and to staff and visitors about their “numb feet” and “bad legs” in a shared vernacular common to those with aged and frail bodies who are experiencing the cumulative chronicity of diseases (Warren et al., 2013). From arthritis to diabetes, dementia, and Parkinson’s, most residents experienced limited movement. Mrs. Casey, aged in her late 70s, had diabetes, and this condition led to sensory neuropathy; she could not feel her feet, and her legs would wobble and give way under her heavy body weight. Diabetes in its late stages can inhibit movement and, for Mrs. Casey, that meant she experienced pain in her legs when walking and was at risk of falling. Like other residents in the facility, difficulty with moving and a perceived risk of falls changed how she could live her everyday life.

Mrs. Casey was effectively confined in her room. She rarely saw or talked to anyone other than staff who delivered meals and pills. Increasingly bored, sedated, and isolated, she put on more weight and had frequent mood swings. The longer she was alone in her room, the stronger became her desire to walk. Unlike able-bodied young people who move with ease, aged residents struggle to move on their own. “Bent backs” and “sore legs” produced discomfort and risks to walking and standing, yet Mrs. Casey still felt a continual urge to move and pushed herself to do a daily walk. With support from the facility’s integrated care management team, Mrs. Casey was allowed access to certain resources that came under the funding category of mobility care: additional assessments by a physiotherapist, a prescribed walking aid, and training sessions with a physio assistant, as well as the allocation of staff time to assist her to relearn how to walk. Before and after lunch, personal care workers were scheduled to help her stand and supervise her daily walk.

How residents can be assisted to move freely and safely plays a key role in them living well and feeling at home in the nursing homes (Zhang, 2023). Mr. Harris was admitted into the aged care facility due to the progression of his Parkinson’s disease. He felt safer in the nursing home environment, where aid devices and technologies were available and staff readily came to his assistance. Whenever his bodily condition allowed, he wanted to spend time on his feet. Meandering along the halls of the nursing home, he connected with other people and things in many ways: by gripping the handlebar of his walker, by feeling the texture of the carpeted floor, by blinking under the ceiling lights, or by feeling the occasional breeze wafting through the front door. His walking merged in a flow of movements with that of other residents, staff, and visitors, and in this way, Mr. Harris wove himself into the lived fabric of the nursing home.

When walking becomes a routine activity, particular material things, places, and people are regularly and consistently incorporated into the self through sensory extension (Dennis, 2007; Petty, 2021). Sometimes Mr. Harris observed people coming and going, and at other times, he would reach out and engage with them. Walking activates connective and associative processes that can enable residents to be in touch with the world. This notion of sensory extension is central to Latimer and Munro’s (2009) notion of relational extension. It involves establishing person-world connectedness through routine practices and creating a sense of belonging. As a productive and relational activity, walking could create *embodied space* “where human experience and consciousness take on material and spatial form” (Low, 2003, p. 9): Not only residents’ bodily space is extended through lifting the foot off the ground and reaching toward the floor, their perceptual field is also broadened by looking ahead and afar. Residents’ bodies and everyday movements are entwined and enabled in the sociomaterialities of the nursing home.

Aid equipment, technologies, and facilities such as corridors, lounges, and dining areas are incorporated into the living environment to support residents’ movements and maintain their safety. Shared handrails support residents’ efforts to move. Thick carpets on the floors protect them from fall-related injuries. A four-wheel walker, like the one that Mr. Harris used, is designed to help balance and make it easier for residents to get around. In the delivery of good care, as Schillmeier and Domènech (2010) have described, technologies such as call-bell and sensor mat alarm systems are used to facilitate connection between residents and staff. Here, the pressure-sensitive mat (a rubber device embedded with electronic wires) is a fall-prevention device that can alert staff to resident movement and the need for help.

Residents often require a helping hand to move and to walk, making staff assistance a key human resource. It entails reaching out to residents through physical proximity, close contact, and intimate touch. Whether it be the helping hand that helps residents up out of the deep recesses of a “comfort chair” or that grasps the handle of a walker to help guide it, staff labor in the kind of “body work” (Twigg et al., 2011) that requires focus and physical exertion. It calls on their own bodies to move in response to residents’ movements. It necessitates becoming attuned to residents’ bodies—a wobble in the legs, a few accelerated jerky steps, a grimace on the face, or any other signal that might suggest a resident is about to lose their footing.

The complexities of resident movement and their multifaceted interdependencies with other people and things point to the processual nature of walking. Taking a Deleuzian approach, walking is always a becoming-walking in which the aligned steps and interlaced hands of care staff and residents come together as one, co-functioning in an assemblage (Deleuze & Guattari, 1988). Assemblage is a key concept for Deleuze and Guattari (1988). It refers to the becomings that occur when objects, beings, events, processes, policies, and discourses coexist. Responding to residents’ wish to move, the assemblage of resources—staff assistance, aid devices, and technologies—and residents’ own efforts come together in abling assemblages of mobility.

However, residents were not always supported to move, to stand, or to walk as they wished. Providing resources to enable residents to move and protect them from falls and fall-related injuries is an important aspect of service delivery in care facilities. Yet, the ethnographer observed situations where residents were restrained from moving under the “duty of care” relating to fall prevention. An example is that of Mrs. Wilson, a resident with later-stage Alzheimer’s disease. She frequently walked around the facility, sometimes at pace. As her dementia progressed, her gait became erratic, and she would often lose her balance. After successive falls and bruises, she was assigned a “princess chair” in the lounge. This is a pressure-cushioned chair that is designed with a curved backrest to provide extra postural support for residents sitting down for long hours. While it is not ostensibly a method of restraint, the backward tilt of the chair inhibits movement and limits engagement with other residents and staff.

From a policy perspective, restrictive practices are not the right way of doing care. In Australian aged care policy, restraint is defined as any practice, device, or action that interferes with a person’s ability to make a decision or which restricts their free movement (Department of Health and Ageing, 2012, p. 1). Although providers are required to operate with a “no restraints” policy, identifying and preventing restrictive practices in aged care can be challenging. In practice, physical and pharmacological restrictive practices are often used on people with cognitive impairment due to dementia, who exhibit “behavioral symptoms,” such as persistent attempts to stand and to walk, despite impaired balance and frequent falls. Restrictive practices are often justified on the basis that they are safety measures to avoid harm to themselves and others. As a care worker explained, “It’s safer for Mrs. Wilson to remain seated.” Instead of enabling Mrs. Wilson to stay active and to walk, safety was prioritized, and this entailed extended long periods of sitting, only relieved by a repositioning routine in which Mrs. Wilson’s position in the princess chair was moved to prevent pressure sores.

This was also the case with Mr. Harris when his gait deteriorated rapidly due to the progression of his Parkinson’s disease. After being assessed as at “high risk of falls,” Mr. Harris was prescribed a new form of mobility care, the “two-staff-assisted daily walking routine,” and a fall-prevention plan that specifically prohibited him from walking on his own. His walking aid was removed, and all attempts to walk, viewed as potentialities for falls, were monitored via a sensor mat in front of his armchair. Just as Mrs. Wilson was trapped in the princess chair, Mr. Harris was prevented from walking by an assemblage of fall prevention, which became restrictive, disabling, and even inhumane, especially when he could not get to the toilet independently.

Residents’ risk of falling could be mitigated by non-restrictive means, that is, by ongoing supervision and prompt assistance; however, that required provision of much more staff support, which was not available. Hence, fall prevention was prioritized due to an ongoing shortage of staff and lack of funding to recruit more. Recognizing his need for mobility, management introduced defined practices, such as the 10- to 15-minute staff-assisted walking routine, as a gesture to “best possible care” within resource constraints.

## Good Care: For Whom and in What Ways?

The provision of “hands on” care to the level required to support frail aging bodies to move, and to move safely, is increasingly being squeezed out of the Australian aged care sector (Zhang, 2023). As recent critical studies reveal, the notion of “care” is being wielded for political ends in times of crisis (Chatzidakis et al., 2020; Hobart & Kneese, 2020). Those studies describe exponential growth in the neoliberal and corporatized versions of care enacted by institutions as they seek to “increase their legitimacy by presenting themselves as socially responsible” (Chatzidakis et al., 2020, p. 11). These forms of “carewashing” present market-driven “care fixes” (Dowling, 2022) that ignore the systemic failures that undermine care, especially for the most vulnerable. Let us examine the regulatory frameworks that shape the aged care sector in Australia. Long before COVID-19 made the headlines or infiltrated aged care homes, the hands-on care provided by workers was being subsumed by the ever-increasing administrative burden due to accreditation processes, through which care home operators must regularly show that they meet prescribed standards of care.^1^ In the audit culture (Strathern, 2000) of the contemporary Australian nursing home, care routines become tasks that must be itemized and meticulously documented by staff. Care must be accounted for because the accounting of care is critical to remain an accredited provider. Facilities that fail to meet the accreditation standards (e.g., the 1997 Aged Care Act and the Aged Care Quality and Safety Commission Act 2018) lose government funding. Meeting accreditation requirements is thus vital.

Adequate staff-supported resident walking that seeks to enable mobility and reduce the risk of falls remains largely a pipe dream in the existing system because low staffing levels and limited time for each resident keep staff fully occupied with essential care activities like washing and feeding, rather than assisting residents to walk. In her influential work, Mol (2002, 2008) teases out the concept of care, with its multiple meanings and enactments, offering a critical framework to show how conflicting perspectives of care coexist and intersect in practice. For STS scholars, “what care looks and feels like is both context-specific and perspective-dependent” (Martin et al., 2015, p. 625) and often asks for attentiveness to emerging requirements of care in particular situations (Schillmeier, 2017).

In nursing homes, an active policy to prevent falls and resulting harm is critical to meeting quality standards and compliance processes. Falls must be documented, risk assessments undertaken, and prevention strategies implemented by increasingly casualized workers who are “hugely overstretched, vulnerable, and less able to care” (Chatzidakis et al., 2020, p. 2). Consequently, restrictive practices such as those outlined above are used in the interests of fall prevention, while residents’ personal desires are largely ignored. Prolonged sitting was encouraged in the case of Mrs. Wilson by providing the princess chair, while Mr. Harris’s walking was severely restricted by removing his four-wheel walker and installing a sensor mat.

As the accreditation process focuses on monitoring compliance rather than quality improvement (Productivity Commission, 2011, p. 101), service providers choose to foreground falls risk rather than the more hidden risks of isolation and inactivity, in order to reduce their own risk of liability. This obligation to keep residents safe, described as “duty of care,” is enacted at all levels: in staff training programs and in internal communications, such as handovers or memos, resulting in care practices that reduce risk by limiting resident movement.

Movement itself is thus curtailed in the name of care. Certain outcomes, such as low incidents of falls and injuries, might look good on paper and appear to provide residents with safety and comfort but certainly cannot make residents feel good when they are restrained from moving and unable to do the things they want to do. In care homes, staff assistance is itemized as service provision through hourly-paid labor. Staff are rostered and remunerated for shift work that is recorded as hours worked. Thus, the availability of staff assistance is always timed and limited by funding levels. Unlike the “abling” assemblages of mobility care described above, which rely on staff time and labor, the “disabling” assemblages of fall prevention can be implemented with minimal resources: a 10- to 15-minute staff-assisted walk or the repositioning of someone sitting in a princess chair. But this kind of care failed to meet the often-unspoken needs of residents who wanted to keep moving and maintain connection with others (Zhang, 2023).

Even before the emergence of COVID, the aged care sector in Australia was in crisis. In its final report, the Royal Commission into Aged Care Quality and Safety^2^ noted that care, as provided in the existing system, cannot assist older people to live a good quality of life (Pagone & Briggs, 2021). The report identified that the fundamental systemic flaw with the Australian aged care system is its focus on the funding requirements of care providers rather than the care needs of older people. In response to this institutional failing, and the proliferation of cases of abuse and neglect, there are calls for fundamental reform of the aged care system to place aged people themselves at the center of aged care provision.

Aged Care Quality Standards were introduced in July 2019 and designed to shift the rhetoric from “ticking boxes” to giving older people control over their care by tailoring care to their individual needs (Aged Care Quality and Safety Commission, 2019). Unlike the old Accreditation Standards that focus on the outcomes for providers, the new Quality Standards center residents and their families as “consumers of aged care services” (Aged Care Quality and Safety Commission, 2019) in a deliberate shift toward a consumer choice framework.

Reframing the aged care sector into a market-led for-profit service industry occurred in tandem with the co-contribution funding model, launched as part of aged care reform in 2012. These changes transformed what was once a heavily government-subsidized sector. Reflecting a neoliberal policy agenda, older people and their families became “customers,” seen as responsible for making choices about what they wanted and how much they would pay for it. Thus, the government implicitly transferred its responsibility for residential aged care to senior Australians themselves, their families, and service providers.

In her book, *The logic of care: Health and the problem of patient choice*, Mol (2008) argues that the mantra of individual choice undermines ways of thinking and acting crucial to health care. Using examples from diabetes clinics and diabetes self-care, she argues that good care is not a matter of individuals making well-argued choices but is something that emerges when all parties negotiate and collaborate to attune medical knowledge and technology to the diseased bodies and complex lives of patients. Unlike the diabetic patients in Mol’s study, who were mostly able to self-care, frail aged care residents rely on staff physical assistance to support all their daily activities, from washing to walking. In this context, good care does come in the form of knowledge and technologies but also, quite critically, from bodily presence and attendance. Care workers align their own minds and bodies with those of residents in order to respond to needs and wishes (Driessen, 2018). If care is seen in practical terms—in walking, with or without staff standby; in sitting, in a chair that is set back or upright; in lending a hand or supplying an assistive device to enable residents to walk safely—the rhetoric of individual responsibility embedded in the phrase “consumer choice” is irrelevant, if not incongruous. Daily walking routines are less a matter of individual choice but a complex set of care relations that are implicated in the accomplishment of feeling at home (Schillmeier, 2014) through walking. To paraphrase Puig de la Bellacasa (2011), they are a matter of good care. Good care, as voiced by residents and staff and supported by fieldwork observations, is more about enabling older people to move and to do the things they would like to, but are unable to do on their own, and assembling human and material resources to do so.

After years of neoliberal approaches to aged care provision, providers are increasingly understaffed and underfunded. With limited access to training and resources in the current system, underpaid staff are often overwhelmed and out of their depth (Pagone & Briggs, 2021, p. 78). The next section extends the discussion on the politics of good care to considering what happened in Australian residential facilities during the COVID-19 pandemic. We will reveal how staff shortages, increased workloads, and insufficient funding during COVID further dismantled any potential for abling assemblages.

## “Back to Basics”: Short-circuiting Care During COVID-19

We know that care that enables residents to move is often not available to them when they need it (Kontos et al., 2011), and even before the pandemic, good care emerged not because of the system but in spite of it. In the nursing homes, staff employed tactics of *la perruque*—not to benefit themselves but to assist the residents whom they were employed to care for (De Certeau, 1984). This meant borrowing time from one resident, whenever possible, to respond to another resident’s immediate needs. During the pandemic, however, residents and staff found themselves in ever more challenging situations where their already limited access to resources was further cut back. In this section, we turn to the emergence of COVID-19 in Australian aged care homes to examine its impact on policies, daily practices, and material surroundings, and we analyze how, when residents’ access to many resources was strictly limited, the abling assemblages that supported them to move were severely ruptured.

Transmission of COVID both affects and results from the multifaceted interdependencies between residents, care workers, and assistive devices already described in this article. Assisting residents with daily living activities means that shaky hands, supportive touch, and a stable walking aid come together in physical proximity, creating intimate circuits of care through which the COVID-19 virus may move easily from one body to another, spreading infection widely. First identified in December 2019, then pronounced a global pandemic in March 2020, the COVID illness can lead to severe acute respiratory syndrome coronavirus 2. Prior to effective treatments or vaccines, public health interventions were vital to combat the spread of infection and mortality. Stopping the transmission of COVID entails monitoring and restricting the interactions of people and their bodily fluids. When exhaled by an infected person, aerosols or droplets containing the virus are inhaled through contact with the eyes, nose, or mouth of another person. People may also become infected by touching contaminated surfaces. It was early understood that minimizing movement decreases the risk of infected people passing the virus to others.

The first COVID outbreak in an Australian residential aged care facility occurred on March 3, 2020, at Dorothy Henderson Lodge in Sydney, and the then prime minister, Scott Morrison, announced restrictions to residential aged care on March 18, 2020. These measures included restricting visitor numbers, physical distancing, and limiting residents’ movements and activities in communal areas (Alderslade, 2020b). In South Australia, the Residential Aged Care Facilities COVID-19 Direction (South Australia Government, 2020) was enacted in aged care facilities on August 13, 2020. Under this protocol, providers were required to implement precautionary restrictive measures including density management and physical distancing in all communal areas, that is, the spaces where people (residents, staff, or visitors) and things (handrails, call-bells, furniture) come into contact with each other. As the risk of transmission increases once the “density,” or “maximum number” of people in a single area, is reached, access to communal areas was reduced. Residents’ bodies, thus, became quantified, with their movements documented and monitored. Providers were also required to implement other infection-control measures such as screening staff and visitors for COVID-like symptoms and regular cleaning of shared surfaces. Communal areas were now regarded as labor-intensive sites of infection control to which residents’ access must be restricted.

As described earlier, prior to COVID-19, residents in this study often reduced their sense of spatial and social isolation by venturing beyond the confines of their individual rooms. Stepping outside their doors, they came into contact with shared handrails in the corridors and carpeted pathways that led to tables and chairs in the lounge and dining areas where they could commune with other persons. Movements they experienced in those outings consisted of helpful assemblages of grab rails, walking aids, and staff assistance, all supporting their attempts to move. However, communal spaces, where residents could overcome their bodily restrictions through spatial extension, were now, for the most part, out of bounds. The previously productive elements that enabled resident movement, such as a hand supporting a resident’s back when they walked, were now classed as hazardous routes of transmission.

Policy directives emphasizing infection control served to amplify the climate of risk aversion among aged care providers, who responded to legal and policy requirements with excessive restrictive practices—often exceeding recommendations. Nationwide, many aged care facilities decided to ramp up and enforce a total lockdown, closing their doors to family and friends, and confining residents to their rooms. This happened even among facilities that had not suffered outbreaks (Alderslade, 2020c). Thousands of residents in homes without COVID-positive cases endured months of isolation in their individual rooms. According to the national Communicable Diseases Network Australia (2021), this was not justified. Residents were in effect “grounded” in a disabling “environment that [was] unresponsive to the needs and aspirations of people with disability” (Matereke, 2020, p. 88), and residents were doubly, or even triply, burdened and excluded: imprisoned in a “comfort chair” and shut in their individual room, which became their lived experience in care.

In a special report^3^ on COVID (Pagone & Briggs, 2020), the Australian Royal Commission into Aged Care Quality and Safety tells the story of a daughter (who they name UY) and her father, who was a nursing home resident with motor neurone disease. He was non-verbal and relied on physical touch to communicate. His facility in New South Wales went into lockdown in March 2020 due to COVID. This meant that UY could no longer hug or touch her father or hold his hand while going for a walk in the gardens of the facility. UY told the commission that her father could not understand why he could no longer touch and hug his family: His health deteriorated rapidly, and he died. UY believed her dad had an innate need for connection and that he deteriorated because it was denied to him. As UY stated,

A nursing home can never be what a family is to someone. It will never fill the gap, but it is a tool to help families with their loved ones. It will never replace the love and connection a family can give to loved ones, and it should not assume that it has the right and authority to do that. (Pagone & Briggs, 2020, p. 7)

The impact of COVID-19 on aged care policies ruptured the abling assemblages that formerly allowed residents to move and severed their vital connections with the outside world.

Restraint on resident movement and restriction of visits from family or friends has had tragic, irreparable, and lasting ill effects. So why did nursing homes enforce restraint and restriction, even in the absence of confirmed COVID-19 cases? Known critical shortages of care staff and personal protective equipment (PPE) in residential facilities during the pandemic might offer a clue in an answer to this question. We contend that it was not the risk of infection but the lack of human and material resources that underpinned the draconian policy of restraint and restriction that was enforced in aged care homes in 2020 to 2021.

Implementing infection control in addition to daily care delivery requires higher staffing levels. To allow movements of residents and visitors within facilities in ways that are COVID-safe, a set of transmission-based precautions need to be integrated into daily care routines. Having care workers dressed in appropriate PPE, such as a mask, apron, and gloves, can reduce the risk of close-contact transmission while assisting residents in physical proximity. Allocating sufficient staff for regular surface cleaning of the tables, chairs, and handrails could mitigate the risk of transmission while still allowing access to these areas. In addition to measures of density management and symptom screening, the implementation of infection control and the provision of daily care may achieve a balance to create COVID-safe assemblages that enable residents to move more freely despite the restrictions on movement. In short, additional staff, skills, and resources are required to provide care safely during an outbreak of COVID.

However, the 2022 Senate Select Committee on COVID-19 inquiry into how the government responded to the outbreak found that residential aged care providers were forced to operate with inadequate resources. Already in crisis in the lead up to the pandemic, the aged care system depends on an increasingly casualized workforce. Women and those from minority ethnic and migrant backgrounds (Department of Health, 2020) are overrepresented in an understaffed, de-professionalized, and demoralized workforce (Hodgkin et al., 2017). Since COVID, the Australian Government announced the allocation of A$500 million to the sector, yet this assistance has not materialized into more hands delivering care. A national survey conducted by the Australian Nursing and Midwifery Federation found that 80% of survey participants reported no increase in care staff to prepare for a potential COVID-19 outbreak (Alderslade, 2020a). Without sufficient government support, the impact of inadequate staffing, poor infection-control measures, and PPE shortages combined to produce an extreme vulnerability to COVID-19 within the aged care sector (Buhler et al., 2021).

Aged care workers were required to carry on with “business as usual” despite insufficient supplies of PPE to protect themselves or the residents (Pagone & Briggs, 2020). On one independent aged care website, a staff member reported, “I understand there are limited supplies of PPE, but I still believe that we aren’t being given a fair opportunity to protect ourselves and our other residents that we have to care for” (Alderslade, 2020a). The secretary of the Victorian Health Workers Union expressed their view to the commission that union members “right now feel like they’re [down] at the bottom of the Titanic” (Pagone & Briggs, 2020, p. 25). In another blunder, aged care residents and staff were not prioritized in Australia’s vaccination effort; limited supplies exacerbated delays in the vaccine rollout (The Senate Select Committee on COVID-19, 2022, p. 62) and made aged care residents and those who care for them more vulnerable to the virus and to increased restrictions to movement.

Two years into the pandemic, the ensuing Omicron wave has further deepened the crisis in Australia’s aged care sector, with staffing fallen to an all-time low. Care workers are pushed to their limit to make sure that even “basic care” gets done (Tooby, 2022). “Basic” refers to daily activities under the “personal care” funding category, including eating, drinking, toileting, and washing. These practices were deemed fundamental to the maintenance of life, but activities that fell under other funding categories, such as staff-assisted walking (under mobility care) or social lifestyle activities (e.g., coffee and chat), were classed as “non-essential” and “high risk” and curtailed. Thus, fundamental elements of care were scrapped, reducing the potential for residents to reconfigure their declining bodies and capacities through sensorial extensions and amalgamations. In the words of an assistant nurse working in an aged care facility in New South Wales, staff cannot sometimes even provide “the basics” to its residents, such as a shower (Davey, 2022). Good care requires “tinkering” (Mol et al., 2010) with a multifaceted assemblage of people and things. It requires resources, especially in the form of human assistance. And it takes significantly more time and energy for staff to conduct infection-control measures on top of their regular care routines.

Going “back to basics” was a market-driven “care fix” (Dowling, 2022) to aged care staff shortages, increased workloads, and insufficient funding, exacerbated by the impact of the COVID-19 pandemic. Ongoing monitoring of resident and visitor movement, as well as the constant screening, cleaning, and management of bodies, surfaces, and buildings to prevent virus transmission, increased staff workloads significantly. In this context, the prioritization of restrictive measures served to decrease the workloads of already overburdened staff.

## Conclusion

There has always been disproportionate “risk and liability” associated with nursing home residents’ right to move. COVID-19 amplified the risk discourse and exacerbated neoliberal care agendas. Infection-control measures locked residents out of communal areas, instructed them to “stay in their personal rooms,” and restricted visits from families and friends. Care facilities appeared to prioritize residents’ safety but cast them into a “triple jeopardy” (Shakespeare et al., 2021). This increased their risk of poor mental health outcomes, reduced their access to care, and amplified the adverse impacts of social and physical isolation. Meanwhile, undervalued and underpaid aged care workers, required to work at the front lines of the pandemic, experienced increased exhaustion, depression, anxiety, and stress (Adelson et al., 2021). Their precarious position of being unable to afford to work at only one site (as mandated), or stay at home while sick, only rendered them and their residents more vulnerable to contracting coronavirus.

This article reveals how resident mobility is contingent on relational interdependencies between residents and those who care for them and how restricting movement can disable resident mobility and rupture their social worlds. Zhang’s experience of walking and moving with residents, particularly for those with physical and cognitive decline, offers grounds for rethinking the issue of restriction, both in relation to the spread of the virus and to movement in general as it applies to residents and staff in aged care facilities. In this article, we propose the need for alternative interventions that prioritize valuing the sensory and social worlds in which residents experience their lives. This involves a shift from previous policy emphasis on eliminating risks of infectious disease toward a relational approach that emphasizes the embodied interdependencies of residents and staff and addresses the structural inequities of contemporary aged care provision.

To support residents to exercise their right to move, we call for new interventions that enable relational approaches to care (Kontos et al., 2017), especially in times of crisis when access to resources is constrained. Governments and care providers all have a responsibility to ensure that in a pandemic, there are (adequately renumerated) staff available to develop strategies that are ethically attuned with residents’ needs for freedom of movement. Facilities do not need to apply severe restraint if they can offer sufficient individual hands-on care. We also call for policies that address the structural inequalities of the aged care workforce. Aged care work is physically, mentally, and emotionally demanding, but care staff are underpaid and overworked. In times of crisis, aged care facilities require more care, not less. It is only by taking a multifaceted approach with heterogenous elements and practices, with supportive infrastructure and increased numbers of well-trained and paid staff, that good care can be assembled.

## Notes

1. The Accreditation Standards (no longer followed after July 2019) formed a part of the Quality of Care Principles 2014. There were four standards, and each standard included a principle and several expected outcomes. The performance of each residential facility would be assessed by the Australian Aged Care Quality Agency against 44 expected outcomes across the four standards to be eligible to receive subsidies from the Australian Government.

2. The Royal Commission into Aged Care Quality and Safety was established on October 8, 2018, by the Governor-General of the Commonwealth of Australia to examine the quality of aged care services in Australia and to judge whether those services are meeting the needs of the community.

3. This report was the result of a hearing of the Australian Royal Commission into Aged Care Quality and Safety on the impact of COVID on aged care, which was held in Sydney from August 10 to 13, 2020.

## References

[bibr1-12063312231159227] Adelson P. Fish J. Peters M. Corsini N. Sharplin G. Eckert M. (2021). COVID-19 and workforce wellbeing: A survey of the Australian nursing, midwifery, and care worker workforce. https://www.unisa.edu.au/contentassets/0429d3a6ea70464b80a0b37aa664aa0c/covid-19-and-workforce-wellbeing-survey_report_final.pdf

[bibr2-12063312231159227] Aged Care Quality and Safety Commission. (2019). Quality standards consumer resources. https://www.agedcarequality.gov.au/consumers/standards/resources

[bibr3-12063312231159227] Alderslade L. (2020a). COVID-19 and what you need to know about protective equipment. https://www.agedcareguide.com.au/talking-aged-care/covid-19-and-what-you-need-to-know-about-protective-equipment

[bibr4-12063312231159227] Alderslade L. (2020b). Prime Minister announces further restrictions to aged care. https://www.agedcareguide.com.au/talking-aged-care/prime-minister-announces-further-restrictions-to-aged-care

[bibr5-12063312231159227] Alderslade L. (2020c). Prime Minister delivers an ultimatum to aged care sector. https://www.agedcareguide.com.au/talking-aged-care/prime-minister-delivers-an-ultimatum-to-aged-care-sector

[bibr6-12063312231159227] Buhler C. Wali N. Ball C. Gurung S. Schismenos S. (2021). The aged care crisis in Australia’s COVID-19 success story. A commentary. Humboldt Journal of Social Relations, 43, 51–59. https://www.jstor.org/stable/48638660

[bibr7-12063312231159227] Chatzidakis A. Hakim J. Littler J. Rottenberg C. Segal L. (2020). From carewashing to radical care: The discursive explosions of care during Covid-19. Feminist Media Studies, 20(6), 889–895. 10.1080/14680777.2020.1781435

[bibr8-12063312231159227] The Communicable Diseases Network Australia. (2021). CDNA national guidelines for the prevention, control and public health management of COVID-19 outbreaks in residential care facilities in Australia. https://www.health.gov.au/sites/default/files/documents/2022/02/cdna-national-guidelines-for-the-prevention-control-and-public-health-management-of-covid-19-outbreaks-in-residential-care-facilities-in-australia.pdf

[bibr9-12063312231159227] Davey M. (2022, February 4). ‘Yelling out for help’: The atrocious conditions inside Australia’s aged care homes. The Guardian. https://www.theguardian.com/australia-news/2022/feb/05/yelling-out-for-help-the-atrocious-conditions-inside-australias-aged-care-homes

[bibr10-12063312231159227] De Certeau M . (1984). The practice of everyday life ( Rendall S. , Trans.). University of California Press.

[bibr11-12063312231159227] Deleuze G. Guattari F. (1988). A thousand plateaus: Capitalism and schizophrenia ( Massumi B. , Trans.). The Athlone Press.

[bibr12-12063312231159227] Dennis S. (2007). Police beat: The emotional power of music in police work. Cambria Press.

[bibr13-12063312231159227] Department of Health. (2020). The 2020 aged care workforce census report. https://www.health.gov.au/sites/default/files/documents/2021/09/2020-aged-care-workforce-census.pdf

[bibr14-12063312231159227] Department of Health and Ageing. (2012). Decision-making tool: Supporting a restraint free environment in residential aged care.

[bibr15-12063312231159227] Dowling E. (2022). The care crisis: What caused it and how can we end it? Verso Books.

[bibr16-12063312231159227] Driessen A. (2018). Sociomaterial will-work: Aligning daily wanting in Dutch Dementia care. In Krause F. Boldt J. (Eds.), Care in healthcare: Reflections on theory and practice (pp. 111–133). Springer. 31314350

[bibr17-12063312231159227] Hobart H. J. K. Kneese T. (2020). Radical care: Survival strategies for uncertain times. Social Text, Social Text, 38(1), 1–16. 10.1215/01642472-7971067

[bibr18-12063312231159227] Hodgkin S. Warburton J. Savy P. Moore M. (2017). Workforce crisis in residential aged care: Insights from rural, older workers. Australian Journal of Public Administration, 76(1), 93–105. 10.1111/1467-8500.12204

[bibr19-12063312231159227] Kontos P. C. Miller K. L. Kontos A. P. (2017). Relational citizenship: Supporting embodied selfhood and relationality in dementia care. Sociology of Health and Illness, 39(2), 182–198. 10.1111/1467-9566.12453 28177149

[bibr20-12063312231159227] Kontos P. C. Miller K. L. Mitchell G. J. Cott C. A. (2011). Dementia care at the intersection of regulation and reflexivity: A critical realist perspective. The Journal of Gerontology B, 66(1), 119–128. 10.1093/geronb/gbq022 PMC3797086 20375084

[bibr21-12063312231159227] Latimer J. Munro R. (2009). Keeping & dwelling: Relational extension, the idea of home, and otherness. Space and Culture, 12(3), 317–331. 10.1177/1206331209337565

[bibr22-12063312231159227] Low S. M. (2003). Embodied space(s): Anthropological theories of body, space, and culture. Space and Culture, 6(1), 9–18. 10.1177/1206331202238959

[bibr23-12063312231159227] Martin A. Myers N. Viseu A. (2015). The politics of care in technoscience. Social Studies of Science, 45(5), 625–641. 10.1177/0306312715602073 26630814

[bibr24-12063312231159227] Matereke K. (2020). Mobilizing disability studies: A critical perspective. Transfers, 10(1), 86–99. 10.3167/TRANS.2020.100110

[bibr25-12063312231159227] Mol A. (2002). The body multiple: Ontology in medical practice. Duke University Press.

[bibr26-12063312231159227] Mol A. (2008). The logic of care: Health and the problem of patient choice. Routledge.

[bibr27-12063312231159227] Mol A. Moser I. Pols J. (Eds.). (2010). Care in practice: On tinkering in clinics, homes and farms. Columbia University Press.

[bibr28-12063312231159227] Pagone T. Briggs L. (2020). Aged care and COVID-19: A special report. https://agedcare.royalcommission.gov.au/sites/default/files/2020-10/aged-care-and-covid-19-a-special-report.pdf

[bibr29-12063312231159227] Pagone T. Briggs L. (2021). Care, dignity and respect. https://agedcare.royalcommission.gov.au/sites/default/files/2021-03/final-report-volume-1.pdf

[bibr30-12063312231159227] Petty K. J. (2021). Beyond the senses: Perception, the environment, and vision impairment. Journal of the Royal Anthropological Institute, 27(2), 285–302. 10.1111/1467-9655.13490

[bibr31-12063312231159227] Productivity Commission. (2011). Caring for older Australians: Overview (Report No. 53).

[bibr32-12063312231159227] Puig de la Bellacasa M. (2011). Matters of care in technoscience: Assembling neglected things. Social Studies of Science, 41(1), 85–106. 10.1177/0306312710380301 21553641

[bibr33-12063312231159227] Schillmeier M. (2007). Dis/abling practices: Rethinking disability. Human Affairs, 17, 195–208. 10.2478/v10023-007-0017-6

[bibr34-12063312231159227] Schillmeier M. (2014). Eventful bodies: The cosmopolitics of illness. Ashgate.

[bibr35-12063312231159227] Schillmeier M. (2017). The cosmopolitics of situated care. The Sociological Review, 65(2_suppl), 55–70. 10.1177/0081176917710426

[bibr36-12063312231159227] Schillmeier M. Domènech M. (Eds.). (2010). New technologies and emerging spaces of care. Ashgate.

[bibr37-12063312231159227] The Senate Select Committee on COVID-19. (2022). The Senate Select Committee on COVID-19 final report. https://parlinfo.aph.gov.au/parlInfo/download/committees/reportsen/024920/toc_pdf/Finalreport.pdf;fileType=application%2Fpdf

[bibr38-12063312231159227] Shakespeare T. Ndagire F. Seketi Q. (2021). Triple jeopardy: Disabled people and the COVID-19 pandemic. The Lancet, 397, 1331–1333. 10.1016/S0140-6736(21)00625-5 PMC7963443 33740474

[bibr39-12063312231159227] South Australia Government. (2020). Emergency management (Residential Aged Care Facilities No. 7) (COVID-19) direction 2020. https://www.covid-19.sa.gov.au/emergency-declarations/aged-care

[bibr40-12063312231159227] Strathern M. (2000). Audit cultures (Vol. 1). Routledge.

[bibr41-12063312231159227] Tooby N. (2022). Omicron worsens crisis in Australia’s nursing homes: Critical care needs of older people unmet during Covid-19 surge. https://www.hrw.org/news/2022/01/30/omicron-worsens-crisis-australias-nursing-homes

[bibr42-12063312231159227] Twigg J. Wolkowitz C. Cohen R. L. Nettleton S. (2011). Conceptualising body work in health and social care. Sociology of Health and Illness, 33(2), 171–188. 10.1111/j.1467-9566.2010.01323.x 21226736

[bibr43-12063312231159227] Warren N. Canaway R. Unantenne N. Manderson L. (2013). Taking control: Complementary and alternative medicine in diabetes and cardiovascular disease management. Health (London), 17(4), 323–339. 10.1177/1363459312460699 23014892

[bibr44-12063312231159227] Zhang R. Y. A. (2023). At home in a nursing home: An ethnography of movement and care in Australia. Berghahn Books.

